# Design and implementation of a Stroke Rehabilitation Registry for the systematic assessment of processes and outcomes and the development of data-driven prediction models: The STRATEGY study protocol

**DOI:** 10.3389/fneur.2022.919353

**Published:** 2022-10-10

**Authors:** Marco Chiavilli, Silvia Campagnini, Teresa Baretta, Chiara Castagnoli, Anita Paperini, Angela Maria Politi, Leonardo Pellicciari, Marco Baccini, Benedetta Basagni, Sara Marignani, Donata Bardi, Alessandro Sodero, Gemma Lombardi, Erika Guolo, Jorge Solano Navarro, Silvia Galeri, Angelo Montesano, Lucia Falco, Marco Giuseppe Rovaris, Maria Chiara Carrozza, Claudio Macchi, Andrea Mannini, Francesca Cecchi

**Affiliations:** ^1^IRCCS Fondazione Don Carlo Gnocchi onlus, Firenze, Italy; ^2^The Biorobotics Institute, Scuola Superiore Sant'Anna, Pisa, Italy; ^3^NEUROFARBA Department, Neuroscience Section, University of Florence, Florence, Italy; ^4^IRCCS Fondazione Don Carlo Gnocchi Onlus, Milan, Italy; ^5^Department of Experimental and Clinical Medicine, University of Florence, Florence, Italy

**Keywords:** stroke, rehabilitation, registry, functional recovery, decision support tools, machine learning, patient-oriented research

## Abstract

**Background:**

Stroke represents the second preventable cause of death after cardiovascular disease and the third global cause of disability. In countries where national registries of the clinical quality of stroke care have been established, the publication and sharing of the collected data have led to an improvement in the quality of care and survival of patients. However, information on rehabilitation processes and outcomes is often lacking, and predictors of functional outcomes remain poorly explored. This paper describes a multicenter study protocol to implement a Stroke rehabilitation Registry, mainly based on a multidimensional assessment proposed by the Italian Society of Physical and Rehabilitation Medicine (PMIC2020), in a pilot Italian cohort of stroke survivors undergoing post-acute inpatient rehabilitation, to provide a systematic assessment of processes and outcomes and develop data-driven prediction models of functional outcomes.

**Methods:**

All patients with a diagnosis of ischemic or haemorrhagic stroke confirmed by clinical assessment, admitted to intensive rehabilitation units within 30 days from the acute event, aged 18+, and providing informed consent will be enrolled. Measures will be taken at admission (T0), at discharge (T1), and at follow-up, 3 months (T2) and 6 months (T3) after the stroke. Assessment variables include anamnestic data, clinical and nursing complexity information and measures of body structures and function, activity and participation (PMIC2020), rehabilitation interventions, adverse events and discharge data. The modified Barthel Index will be our primary outcome. In addition to classical biostatistical analysis, learning algorithms will be cross-validated to achieve data-driven prognosis prediction models.

**Conclusions:**

This study will test the feasibility of a stroke rehabilitation registry in the Italian health context and provide a systematic assessment of processes and outcomes for quality assessment and benchmarking. By the development of data-driven prediction models in stroke rehabilitation, this study will pave the way for the development of decision support tools for patient-oriented therapy planning and rehabilitation outcomes maximization.

**Clinical tial registration:**

The registration on ClinicalTrials.gov is ongoing and under review. The identification number will be provided when the review process will be completed.

## Introduction

Stroke represents the second preventable cause of death after cardiovascular disease and the third global cause of disability (1). More than a quarter of the survivors have a significant disability in the activities of daily living, while half of them suffer from reduced mobility due to hemiparesis (2). In the recent COVID-19 pandemic, the European Stroke Organization warned against the even higher risk of death and disability post-stroke due to the lack of availability of the essential stroke care pathways, with the risk of suboptimal care for affected patients (3). Hence the need for specific, intensive rehabilitation treatment, in the acute and post-acute phases. The rehabilitation of patients with stroke should aim not only at sensorimotor impairment but also at all the problems that this pathology entails, such as pain, depression (4), cognitive, communication, language, and swallowing difficulties, and sphincter and respiratory problems.

In countries where national registries of the clinical quality of stroke care have been established, the publication and sharing of the collected data have led to an improvement in the quality of care and survival of patients (5). However, information on rehabilitation processes and outcomes is often sparse, and predictors of functional outcomes remain poorly explored. According to a definition of the literature, a stroke registry can be defined as “an organized system of collection, storage, analysis and dissemination of data of single individuals who have suffered a stroke” (6). To date, literature data currently document 28 stroke registries in 26 different countries (7). Noticeably, in most cases, the focus of these registries is on the acute care processes and outcomes, and their potential relationship with long term stroke outcomes, while little or no attention is given to rehabilitation (7). The creation of a rehabilitative Stroke Registry stems from the need to create a multidimensional assessment as complete as possible of the patient affected by stroke, and at the same time easily applicable and shared by the whole rehabilitation team in the different care paths and for their entire duration. Among potentially relevant predictors, several lines of research have stressed the importance of features highlighting the conditions of biological fragility and the clinical and nursing burden of intensive rehabilitation inpatients—markers of complexity (8)—in contributing to explain rehabilitation outcomes, regardless of the clinical main diagnosis. Previous researches conducted by our group both on hip fracture (9) and stroke patients (10) seem to confirm that these features provide information that may independently contribute to the prediction of rehabilitation outcomes. Recently, the Italian Society of Physical and Rehabilitation Medicine (SIMFER) has published the updated Minimal Assessment Protocol of a person with a stroke addressing Rehabilitation (PMIC) (11), under the name of PMIC2020 (12). The PMIC project was initiated to provide Italian physiatrists with a common basis for assessing patients with stroke at a given time after onset. The PMIC2020 provides a minimum dataset for the description of the psychosocial, clinical, functional characteristics and outcomes of stroke patients addressing rehabilitation, but its predictive value needs to be verified (12). For this last purpose, the growing tendency toward data-driven and evidence-based rehabilitation is promoting machine learning (ML) applications for diagnosis and the prediction of the post-stroke prognosis. Among prognostic solutions, Zhang et al. (13) aimed at predicting the outcome of treatment at 3 months by analyzing physiological parameters during the first 48 h after stroke using cross-validated logistic regression. In other cases, ML-based solutions were optimized for the prediction of mortality 30 days after the event (14) or at the time of discharge from the acute ward (15). Data-driven prediction may also pave the way for the development of decision support tools for patient-oriented therapy planning and rehabilitation outcomes maximization. However, it is worth noting that currently available solutions in this field are often affected by small sample sizes and validation bias (16).

For these reasons, the IRCCS Fondazione Don Carlo Gnocchi (FDG) in Florence, one of the largest Italian scientific rehabilitation and research institutions (SRRI) has recently developed and implemented an evidence-based interdisciplinary integrated care pathway (ICP) for post-acute stroke inpatient rehabilitation (17) and has designed the STroke RehabilitATion rEgistrY (STRATEGY) Project. STRATEGY will test the feasibility of a Stroke Rehabilitation Registry based on the PMIC2020 and clinical/nursing complexity variables and verify its predictive value using ML-based methods. This work will discuss the design of the Registry contents and will describe its structure and implementation in the Intensive Rehabilitation Units (IRUs) of the FDG centers. The primary outcome of the Registry implementation is to provide a systematic assessment of relevant processes and outcomes in post-acute stroke rehabilitation while verifying its feasibility in clinical practice. The secondary purpose is to use the Registry data to develop and validate predictive models of rehabilitation outcomes in patients with stroke outcomes ML algorithms, as a first step toward the implementation of a Clinical Decision Support System (CDSS) for post-stroke rehabilitation planning.

## Methods

The STRATEGY protocol requires the collection of a series of clinical-functional variables, for a multidimensional, even if minimal, assessment of rehabilitation needs, outcomes, and prognostic factors of stroke patients facing rehabilitation services. The variables were collaboratively discussed within the interdisciplinary study group of the four participant centres, aiming at maintaining the minimum overall feasible evaluation, looking for (1) information variables that can be easily and reliably collected in an inpatient rehabilitation setting, including a follow-up telephone evaluation after the acute event, and (2) internationally recommended and validated tools. Whenever possible, the validated Italian versions were adopted.

### Study design and setting

The study is a prospective, longitudinal, and observational cohort study including the IRUs of the FDG centres, located in Italy. Patients admitted to the IRUs of the participating FDG centres with a diagnosis of ischemic or haemorrhagic stroke within 30 days will be considered for eligibility for this study.

### Participants

All patients admitted to the participating IRUs and fulfilling the following inclusion criteria will be assessed and will be considered eligible for the study. Initially, it has been planned to enroll 600 patients in 30 months (June 2021–May 2024), subdivided into the FDG centres primarily involved: Florence, Milan, Rovato, and Parma. Later, all the FDG centres involved in the FDG National Neuromotor Department and treating stroke patients 9 centres of the FDG have adhered to the study (La Spezia, Turin, Avellino, Ancona, Matera, Potenza, Varese, and Rome with two different centres), thus the final objective is to recruit 1,200 patients in 13 Centers across Italy.

Inclusion criteria:

- First-ever or recurrent ischemic or haemorrhagic stroke
- Diagnosis of Acute Stroke
- Index event within 30 days from admission in IRU
- Age 18+
- Written informed consent.

Exclusion criteria:

- Transitory ischemic attack
- Patients with severe haemorrhagic or ischemic stroke (disorders of consciousness states and critical clinical care conditions), who are addressed to the severe brain injury high-complexity rehabilitation wards.

### Assessment

**Figure 1:**
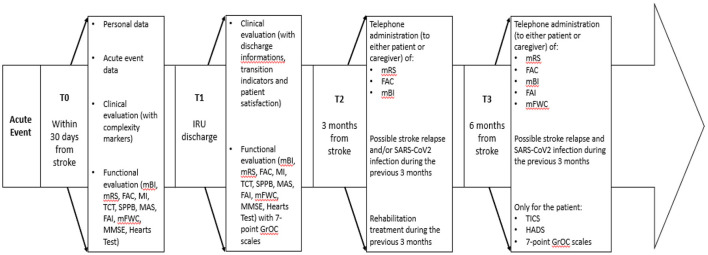
Study timeline: Assessments conducted for each timepoint.

The assessment will be performed at inpatient rehabilitation—admission (T0), inpatient rehabilitation—discharge (T1), and follow-up—three (T2) and six (T3) months after the acute event. The timeline of the study is illustrated in Figure 1.

### Admission evaluation (T0)

Assessment at admission includes a set of variables that can be grouped into four categories: *personal data, acute event data, clinical evaluation*, and *functional evaluation*.

#### Personal data

This category includes the admission setting, the date of admission, patient's mother tongue and level of education, patient's occupation, the possession of civil disability, if present, the presence of a caregiver/administrator to support the patient, the premorbid housing situation, and the housing conditions (presence of family members/cohabitants or not and presence of architectural barriers or not). Measured weight and height and Body Mass Index (BMI) calculation are also inserted in this section.

#### Acute event data

This section includes the date of the acute event, if available, or the date of access to the first aid service. The classification of stroke type (ischemic or haemorrhagic) and subtypes are presented as follows:

- for ischemic stroke, clinical classification of the Oxfordshire Community Stroke Project (18) and etiological classification of the Trial of Org 10172 in Acute Stroke Treatment (TOAST) (19);
- for haemorrhagic stroke, classification by intracerebral or subarachnoid localization.

In addition, this section contains information related to the location of the lesion, the side of the body affected by weakness/paresis, the possible presence of subluxated shoulder and edema of the affected hand, the type of treatment received during the acute phase (thrombolytic therapy, neurosurgical evacuation, etc.), and the complications arising from the procedure, if any (20). Finally, the patient's premorbid functional level according to the modified Rankin Score (mRS) (21) is required.

#### Clinical evaluation

This section includes the presence of associated pathologies/comorbidities, evaluated through the Charlson Comorbidity Index (22), the possible presence of a communication disability, evaluated through the Communicative Disability Scale (SDC) (8), the presence or absence of signs of clinical instability at the time of evaluation, a quantitative assessment of the neurological deficit according to the National Institute of Health Stroke Scale (NIHSS) (23), and the presence or not of complexity markers (altered alertness, delirium, acute infection in progress, depression, pain, dysphagia, malnutrition, presence of nasogastric tube (NGT)/percutaneous endoscopic gastrostomy (PEG), presence of bladder catheter or urinary incontinence, presence of central venous catheter, tracheostomy tube, anemia, or dialysis). Complexity markers belong to the so-called “status indicators,” which define the clinical and functional profile of patients, both at admission and at discharge, highlighting the conditions of biological fragility capable of influencing the rehabilitation course and the outcomes of rehabilitation treatment (8).

#### Functional evaluation

It comprehends the evaluation of autonomy in the activities of daily life using the modified Barthel Index (mBI) (24) and the mRS, and of the patient's motor skills through the Functional Ambulation Classification (FAC) (25), the Motricity Index (MI) (26), the Trunk Control Test (TCT) (27), and the Short Physical Performance Battery (SPPB) (28). This section also includes the quantification of muscle spasticity, using the Modified Ashworth Scale (MAS) (29), and the evaluation of the measures of participation before the stroke through the Frenchay Activities Index (FAI) (30) and the modified Functional Walking Categories (mFWC) (31). The evaluation of the patient's cognitive performance is measured through the Mini Mental State Examination (MMSE) (32) and the Hearts Test from the Oxford Cognitive Screening (33).

### Discharge evaluation (T1)

Assessment at discharge includes clinical evaluation and functional evaluation.

#### Clinical evaluation

It includes the presence or absence, at the time of discharge, of the complexity markers already present in T0, and the quantitative evaluation of the neurological deficit through the NIHSS. It also includes the date of discharge, the destination of discharge (home, other hospitals, protected residence, death), the rehabilitation program carried out (physiotherapy, psychological counseling, robotic rehabilitation, etc.) and any proposal for prescription of orthoses/aids. The degree of patient satisfaction with the rehabilitation treatment as a whole is also recorded in a 10-point Likert-type numerical scale, in which the value 0 corresponds to “not at all satisfied” and the value 10 to “fully satisfied.” This section also includes transition indicators (8), i.e., events that marked the rehabilitation process divided between adverse clinical events (infectious, non-infectious and falls) and critical processes (restraint, treatment with antidepressants, pain treatment, nutritional treatment oral, and artificial nutrition).

#### Functional evaluation

The same assessment tools administered at admission will be readministered at discharge.

In addition, three 7-point (much worse, worse, a bit worse, about the same, a bit better, better, much better) Global Rating of Change (GRoC) scales (34) will be used to measure the self-perceived change in health status, as regards to both independence in the activities of daily life and motor control of paretic limbs.

### Follow-up evaluation (T2 and T3)

Assessment at T2 (3 months after the acute event) includes the telephone administration of the mRS, FAC and mBI. Furthermore, a possible stroke relapse and SARS-CoV2 infection are investigated, and patients are asked whether they have undertaken rehabilitation treatment during the previous 3 months. The interview will be administered to either the patient or the caregiver.

Assessment at T3 (6 months after the acute event) includes the administration of a larger number of items: mRS, FAC, mBI, possible stroke relapse and SARS-CoV2 infection, FAI, mFWC (only if the mBI item 9 is scored >0), 11-point NRS for pain. When the interview is conducted with the patient, a few additional scales will be administered: the Telephone Interview of Cognitive Status (TICS) (35), the Hospital Anxiety Depression Scale (HADS) (36), and the three 7-point GRoCs about the perceived change as described above. Furthermore, we investigate possible social fragility and housing conditions.

### Rehabilitation

The rehabilitation intervention is defined in an ICP based on the American Heart Association/American Stroke Association guidelines (17, 37) and will provide, according to the national requirements, at least 3 h per day of specific rehabilitation including physiotherapy, neuropsychological therapy, and speech and dysphagia therapy, in addition to the assessment and training in the use of aids (20). Physiotherapy may also include robotic rehabilitation according to the individual rehabilitation plan defined by the interdisciplinary team (17). The rehabilitation plan will be based on the assessment at admission and will be adapted to emerging needs at any time during the rehabilitation stay, through systematic weekly team meetings. When indicated, psychological support to the patient and/or family will be also provided.

### Outcome measures

The primary outcome is the improvement of functional ability as measured by the mBI, between T0 and T1, between T0 and T2, and between T0 and T3. Secondary outcomes include cognitive recovery, which will be evaluated by comparing the performances of the MMSE between T0 and T1, and recovery of participation at T3, compared to pre-stroke data, according to the FAI and the mFWC. Other selected rehabilitation outcomes will include the length of stay, adverse outcomes (post-stroke epilepsy, deaths, or discharge to acute care hospital) and differences between functional and clinical indicators recorded at T0 and T1, for changes in sensory-motor impairment (MI, TCT) and ambulation (FAC). Changes in markers of complexity will also be considered. Follow-up outcomes will include all of the above.

### Data collection and management

Clinical data collected will be those in the paper medical record. With the consent of the patient or his/her family member/caregiver/legal guardian, the data will be entered anonymously into a computerized database using REDCap (Research Electronic Data Capture) (38), a web application for building and managing research databases. The use of a dedicated tool allows for robust data collection, data quality checks, and the reduction of missing data. All the users will have private credentials and dedicated roles to access the database, for regulated access to the patients' data. Each patient will be associated with a reference ID and the correspondence between the patient's name and the associated ID will be stored in a secure external file, which can only be accessed by the principal investigators of the Coordinating Centre and the managers of the other centres.

### Data analysis

The statistical analysis of the preliminary data will be carried out using the IBM Corp. Released 2020 software. IBM SPSS Statistics for Windows, Version 27.0. Armonk, NY: IBM Corp.

A descriptive analysis of the main personal, clinical, physiotherapy, and cognitive characteristics of the sample at admission and discharge from hospitalization will be performed. More specifically, the continuous variables will be described using the mean and standard deviation or the median and interquartile range, according to data distribution. The normality will be evaluated by the Shapiro-Wilk test. The categorical variables will instead be described by relative frequencies and percentages. Furthermore, the clinical, physiotherapy and cognitive characteristics will be compared, between admission and discharge. For these comparisons, the *t*-test for paired data or its non-parametric version, the Wilcoxon test, will be used for numerical variables, whilst the McNemar test will be employed for categorical variables.

A further step will involve the testing of machine learning methods for outcome prediction. The STRATEGY database will enable the prospective validation of existing ML algorithms for prognosis prediction in stroke and the development of new solutions. Previously available methods will be tested on STRATEGY data and new solutions will be generated using newly available data only or by updating existing solutions through them. The prognostic factors, with respect to the outcomes, will be screened by univariate logistic/linear regression adjusted by age and gender. Then, linear and logistic regressions will be compared with solutions based on support vector machines, random forests, multi-layer perceptron, or “deep” artificial neural networks. Finally, ensemble learning will be tested, merging classifiers/regressors outputs. The performances of different algorithms in predicting recovery outcomes will be compared in terms of accuracy, F1-score, root mean square error and determination coefficient. To limit the risk of wrong interpretation of algorithm results, nested cross-validations will be implemented. It will allow a proper assessment of the generalization capabilities of the algorithm, or in other words to check its reliability when applied to new patients who were not included in model definition and training. The effects of hyper-parameter tuning and features selection strategies will be tested within cross-validation loops. Finally, to foster interpretability of more complex machine learning methods and provide a patient-specific explanation of predictions, saliency maps and other methods such as Shapley's additive explanations, will be deployed.

## Discussion

The past decade has seen great advances in the treatment of acute phase cerebrovascular disease, but stroke is still causing an increasing number of persons surviving with chronic disabilities worldwide (39). Rehabilitation can effectively reduce the burden of post-stroke disability (40), but there are specific challenges still open for research, especially in defining outcome assessment and personalizing rehabilitation strategies. In fact, the optimization of such strategies is still largely limited by the limited knowledge existing on predictive markers of a favorable outcome.

As to outcome definition, we chose to make reference to the PMIC2020. Compared to the original version of PMIC, PMIC2020 brought some innovations, introducing the NIHSS, which provides a more in-depth assessment of stroke-related disability (41) and continuous monitoring of the patients over time, being used also in acute phase settings. Other innovations of PMIC2020 were the substitution of the Barthel Index with the mBI (24), and the assessment of participation, according to a multidimensional bio-psycho-social approach to stroke survivors. Specifically, the FAI was adopted and the Walking Handicap Scale was substituted with the mFWC in the Italian validated version (30). In STRATEGY, we have decided to maintain the variables previously introduced in PMIC2020, and at the same time, we searched for introducing other informative variables that were already reported in the literature as potential predictors of rehabilitation outcomes. We also paid attention to the inclusion of tools that could be easily and reliably collected, aiming to create a comprehensive and feasible assessment for stroke patients addressing inpatient rehabilitation units.

For what concerns spasticity assessment, we chose to integrate the spasticity assessment provided by the PMIC2020 (yes/no for each articular district required in the MAS), with the grading of spasticity required to complete the MAS, as this is the most commonly used scale for assessing limb muscle tone. Spasticity affects ~42% of stroke patients and 15% develop severe spasticity (42). This clinical condition, by causing significant disability, has a considerable impact on activities of daily living, social activities, and the psychological wellbeing of the patient (43). At the same time, spasticity, especially in the lower extremities, plays a major role in walking in patients with severe hyposthenia. For these reasons, the assessment of muscle tone becomes crucial for health professionals. MAS, besides being easy to apply, has shown moderate to high reliability in previous studies (44). Today it represents the gold standard for the assessment of limb spasticity and the evaluation of the effectiveness of rehabilitation and pharmacological intervention (29).

Cognitive disturbances can precede, but also be a direct consequence of stroke, and their frequency must be taken into consideration (45). Cognitive impairment has been correlated with limited functional gains and poor rehabilitation outcomes, especially in elderly patients (46). Rehabilitation is an active process involving the ability to follow, learn, and remember specific instructions, and therefore it is not surprising that patients with lower cognitive scores are less likely to have a favorable outcome. The evidence in the literature also underlines the importance of cognitive wellbeing to benefit from rehabilitation (47, 48), demonstrating that patients with better cognitive conditions benefit more from the rehabilitation treatment and their length of hospitalization is shorter (46). This led researchers to call for cognitive assessment as an integral part of rehabilitation (49). In STRATEGY, according to the PMIC2020, the MMSE has been chosen for the cognitive screening. This decision was supported by the results of a recent review focused on the diagnostic accuracy of cognitive screening tests for detecting post-stroke cognitive impairment (50). From the comparison between the Montreal Cognitive Assessment (MoCA) and MMSE, the review concluded that MoCA has higher sensitivity but lower specificity, and both tests are appropriate. Nevertheless, the Authors highlighted the lack of research on the diagnostic accuracy of the MoCA in a post-acute phase, making the MMSE more suitable in a rehabilitation setting. Moreover, MMSE requires less time to complete administration (on average <10 min) compared to the other test. Since the MMSE does not apply to telephonic interviews, as a screening tool for a telephonic follow-up evaluation we chose the Italian TICS (51) a valid, and reliable, telephone-based cognitive screening test, designed so that its scores can be compared to MMSE (52).

The ability to communicate is considered a highly relevant issue in assessing patients' rehabilitation needs (53), as well as in predicting rehabilitation outcomes (8, 9). Since PMIC2020 did not include a measure for disability on communication, except for the NIHSS items on aphasia and dysarthria, we chose the Communicative Disability Scale (SDC) (8). The SDC evaluates difficulties in communication as assessed by the clinician after an anamnestic interview and clinical examination. The scale was designed for the rapid assessment of any impairment in the communication of patients admitted to rehabilitation settings. It is based on the general criterion of the “burden” of the communicative exchange: the more serious the communication impairment, the greater the burden that the interlocutor will have to bear in the communicative exchange. It does not explain the specifics of the disorders, which can be caused by aphasia, apraxia, dysarthria, dementia, or sensory deficit such as deafness, but the impact on the effectiveness of the communicative exchange.

Preliminary studies (17) suggest that the response to intensive rehabilitation hospitalization in rehabilitation facilities is also strongly dependent on a series of markers of clinical complexity care. These may arise during hospitalization, regardless of the pathology directly related to the acute disabling event. Rehabilitation outcomes surely depend on the intervention of specific impairments, but it also appears that the concurrent resolution of syndromic problems, typical of complex patients (immobility, medical instability, delirium, malnutrition, depression, pain, communication disorders, comorbidities, social fragility, incontinence), plays a fundamental role in achieving a good functional outcome (8, 54). In literature, these conditions are often referred to as Geriatric Syndromes because they occur, often associated, with the frail elderly, although they are not exclusive to elderly patients. The resolution of the syndromes represents itself an outcome of rehabilitation, regardless of the improvement of the patients' overall functional status. The frequency of medical complications in rehabilitating stroke patients varies from study to study depending on patient selection, the type of rehabilitation setting, and the criteria used to define a “medical complication” (54, 55). Among them, another important predictor of the rehabilitation outcome is the assessment of mood. Depression is a common and serious complication after stroke, predicting worse functional outcomes and worse quality of life, but it often goes undiagnosed, and treatment is not consistently provided in common clinical practice (56). Previous studies of post-stroke depression reported prevalence rates ranging from 25 to 79% (57). The introduction of systematic control and assessment of mood can increase the physiatrist's awareness of this condition when visiting stroke survivors. For the purpose of maintaining feasibility also in the outpatient rehabilitation setting, the PMIC2020 opted for a simple definition of mood as adequate/deflected/not evaluable, based on what emerged from the anamnestic data and direct clinical evaluation. In STRATEGY depression has been selected as one of the aforementioned markers of clinical complexity care, defined by a specific algorithm including clinical evaluation AND/OR certified psychiatric diagnosis AND/OR current antidepressant medication (8). Since depression is a common complication of stroke also in the long term, impacting both activities and participation (20), for the follow-up evaluation we opted for the HADS (58). HADS is one of the most frequently used tools for detecting the two distress expressions, i.e., anxious and depressive states, generally accepted by users (59, 60) and often adopted for telephone interviews. The introduction of systematic control and assessment of mood can increase the physiatrist's awareness of this condition when visiting stroke survivors.

Patients with stroke often have other chronic diseases, making comorbidity common in stroke. Patients with multimorbidity have complex health needs and there is the risk that the care they received might be incomplete, fragmented and sometimes ineffective (61). For these reasons, we aim to better understand the relationship between stroke rehabilitation and comorbidities aiming at patient-centred care and thereby improving the patient experience (62). In the PMIC2020, for reasons of brevity, it was chosen to maintain only the presence/absence of comorbidities potentially affecting rehabilitation. To provide a more objective measure of comorbidity, we chose to monitor the concomitant clinical conditions also through the Charlson Comorbidity Index (22), because this is a validated method developed for classifying prognostic comorbidity.

Although anamnestic participation is rarely measured at admission to stroke rehabilitation units, it is now universally recognized that a post-stroke evaluation cannot be considered complete if it does not include an analysis of participation, defined by ICF as an “involvement in a life situation.” In fact, it provides fundamental information to correctly define the objectives of the rehabilitation project because it offers a contextualized picture of the person in his/her environment, made up of interests, attitudes and potential, so much to be considered a major outcome of successful rehabilitation (63). For this purpose, we included a transcultural validated Italian version (30) of the FAI that contains 15 items or activities that can be separated into 3 subscales: domestic chores, leisure/work and outdoor activities.

In STRATEGY, the primary outcome measure, i.e., the mBI, has been chosen to reflect the global functional status of patients, while the secondary outcomes highlight the specific impairments (cognitive, motor, etc.) to be addressed in the individual rehabilitation program, with the aim of designing tailored pathways for specific stroke patients' features and needs. With this work, we aim to identify the clinical markers predicting the outcomes at different time points (at discharge and 3 and 6 months after the acute event). A realistic goal statement is mandatory for designing the individual rehabilitation project (64) and more accurate predictions are necessary to share patients' objectives within the team (including patients and caregivers) and intercept and provide specific interventions for those at high risk of worse outcomes.

In our analyses, instead of limiting our assessment to biostatistics for prognostic markers analysis, we directly target the validation of ML-based methods for a prognostic prediction. Accurate predictions would have a direct application in the clinical practice: they may help rehabilitation planning (e.g., length of stay) as well as verify the efficiency and the effectiveness of rehabilitation intervention. The involvement of multiple centres and the relatively long time of the protocol allow a 2-fold assessment of the robustness of the proposed methodologies. On one hand, the external validation of algorithms will be possible, by assessing the accuracy of a solution developed using data from one site on data from different centres. On the other hand, solutions trained using either retrospective data or data from the initial part of the study will be tested, and eventually updated, using data from the latter part of the study. As a result, thanks to its multifactorial and multicentric assessment, STRATEGY will be the ideal platform to set the basis for the definition of a CDSS for post-stroke rehabilitation.

In conclusion, a Stroke Rehabilitation Registry, including highly informative, easily collected variables in routine clinical practice, is highly needed to provide quality assessment and benchmarking in stroke rehabilitation. Our Stroke Rehabilitation Registry will verify the predictive value of the variables included in the minimal assessment protocol for stroke rehabilitation patients defined by the SIMFER (PMIC2020), along with other potential predictors suggested by previous literature. The STRATEGY database will also provide ground for the development of ML algorithms that may help identify important factors and variables for recognizing risk profiles, predicting treatment success, and, possibly, supporting choices for personalized interventions aimed to optimize rehabilitation outcomes in stroke patients.

## Ethics statement

The studies involving human participants were reviewed and approved by Ethics Committees (Florence: 14513). The patients/participants provided their written informed consent to participate in this study.

## Author contributions

FC, CM, and AMa: study design. MC, AMa, SC, and FC: manuscript draft. CC, AP, MB, LP, and AMP: functional assessment protocol design. BB, SM, and DB: neuropsychological assessment. FC, MC, AS, EG, GL, JN, SG, AMo, LF, MR, and TB: clinical assessment protocol. AMa and SC: statistical analyses. All authors are revision of the manuscript. All authors contributed to the article and approved the submitted version.

## Funding

This study was funded by the Italian Ministry of Health under the Ricerca Corrente RC2020, RC2021, and RC2022 programs and the 5xMille funds AF2018: Data Science in Rehabilitation Medicine AF2019: Study and development of biomedical data science and machine learning methods to support the appropriateness and the decision-making process in rehabilitation medicine.

## Conflict of interest

The authors declare that the research was conducted in the absence of any commercial or financial relationships that could be construed as a potential conflict of interest.

## Publisher's note

All claims expressed in this article are solely those of the authors and do not necessarily represent those of their affiliated organizations, or those of the publisher, the editors and the reviewers. Any product that may be evaluated in this article, or claim that may be made by its manufacturer, is not guaranteed or endorsed by the publisher.
